# The Containment Scouts: First Insights into an Initiative to Increase the Public Health Workforce for Contact Tracing during the COVID-19 Pandemic in Germany

**DOI:** 10.3390/ijerph18179325

**Published:** 2021-09-03

**Authors:** Dunja Said, Simon Brinkwirth, Angelina Taylor, Robby Markwart, Tim Eckmanns

**Affiliations:** 1 Unit 37—Healthcare-Associated Infections, Surveillance of Antibiotic Resistance and Consumption, Department of Infectious Disease Epidemiology, Robert Koch Institute, Seestraße 10, 13353 Berlin, Germany; TaylorA@rki.de (A.T.); EckmannsT@rki.de (T.E.); 2 Institute of General Practice and Family Medicine, Jena University Hospital, Bachstraße 18, 07743 Jena, Germany; Robby.Markwart@med.uni-jena.de

**Keywords:** contact tracing, contact management, SARS-CoV-2, COVID-19, containment scouts, public health workforce

## Abstract

The COVID-19 pandemic in Germany has demanded a substantially larger public health workforce to perform contact tracing and contact management of COVID-19 cases, in line with recommendations of the World Health Organization (WHO). In response, the Robert Koch Institute (RKI) established the national “Containment Scout Initiative” (CSI) to support the local health authorities with a short-term workforce solution. It is part of a range of measures for strengthening the public health system in order to limit the spread of SARS-CoV-2 in Germany. The CSI is an example of how solutions to address critical health system capacity issues can be implemented quickly. It also demonstrates that medical or health-related backgrounds may not be necessary to support health authorities with pandemic-specific tasks and fulfil accurate contact tracing. However, it is a short-term solution and cannot compensate for the lack of existing qualified staff as well as other deficits that exist within the public health sector in Germany. This article describes the structure and process of the first phase of this initiative in order to support health policymakers, public health practitioners, and researchers considering innovative and flexible approaches for addressing urgent workforce capacity issues.

## 1. Introduction

The public health workforce is one of the essential components of a functional health system and is directly linked to its quality [1]. However, underfunding of health systems has become an increasing issue in many European countries [2], which has resulted in a shortage of staff in the public health sector [3]. As a result of the COVID-19 pandemic, health systems worldwide have been placed under even further pressure. The needs and tasks have increased rapidly across the health system, from preventative to acute care services; and many shortcomings, including understaffing, have become visible.

**Figure 1:**
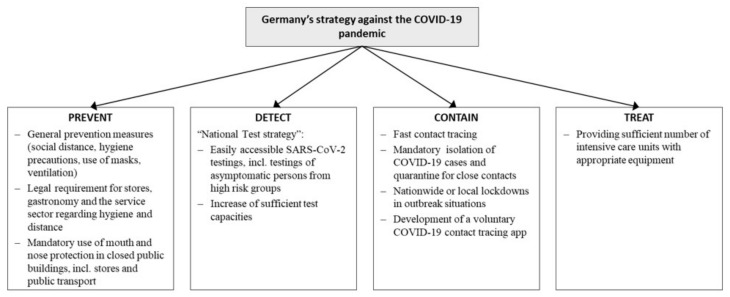
Germany’s technical strategy against the COVID-19 pandemic.

The public health workforce in Germany has been particularly impacted by the pandemic, with limited capacities and resources already prior to the pandemic [4]. In addition, the usual health workforce capacity has been depleted at times due to sickness absence as a result of COVID-19. The German government responded to the pandemic by delivering its pandemic strategy [5] and comprehensively introducing measures at every level of the health system (Figure 1).

The European Centre of Disease Control and Prevention (ECDC) recommended the following set of measures to address the pandemic and contain further outbreaks: physical distancing, hygiene measures, sufficient testing, contact tracing, and appropriate management of contacts [6]. Contact tracing aims to break the chain of transmission through the systematic management of people (termed “contacts”) who have been exposed to an infected person. The main steps involve identifying, assessing, and following up on contacts. The tasks are typically performed by public health and medical professionals [7]. A major recommendation from a World Health Organization (WHO) mission in China in February 2020 was to increase workforce capacities and prioritise case finding through rapid and accurate decentralised contact tracing teams [8].

**Figure 2:**
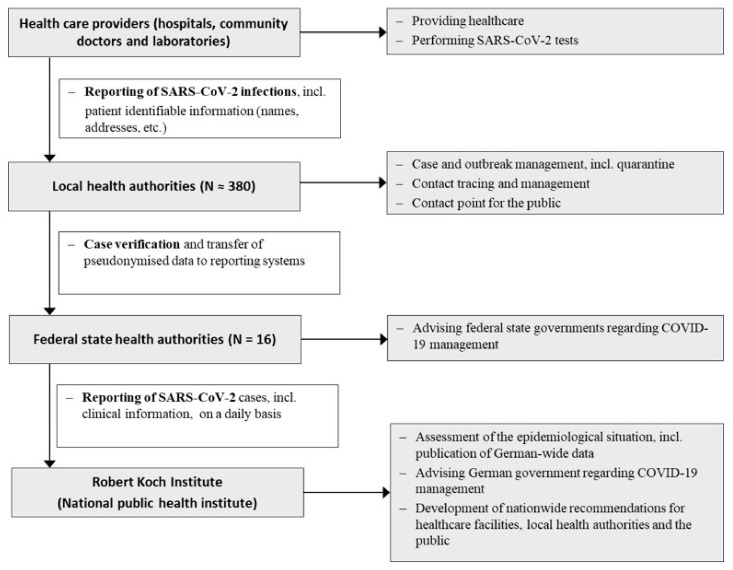
Public health system, outbreak management and reporting system in Germany.

As part of the range of measures to address the pandemic in Germany and in response to ECDC and WHO recommendations, contact tracing and contact management of people with COVID-19 was introduced, one of the most time-consuming and labour-intensive pandemic activities carried out since the start of the pandemic [9]. The public sector alone is responsible for contact tracing in Germany, and the private sector does not play a role. This responsibility is shared by the Robert Koch Institute (RKI), the German national public health authority, which carries out international contact tracing, and the approximately 380 local public health authorities who are responsible for contact tracing within Germany (Figure 2) [10]. In addition to the CSI, additional staff to support contact tracing in Germany have been drawn from other sources, including staff from the army (Bundeswehr), public servants from all state levels, and medical students [7,11,12].

The pandemic has required countries to quickly respond with approaches over the short term, making the pandemic a unique scenario for speed and innovation. The Containment Scout Initiative (CSI) was developed as an innovative approach to address the public health workforce capacity challenge in contact tracing services in Germany. The RKI established the CSI in cooperation with the Federal Office of Administration (Bundesverwaltungsamt, BVA), inspired by the approach implemented in China. The purpose of this paper is to describe the concept and processes of this evolving initiative as well as the results from surveys conducted during the first phase (March 2020 to October 2020) in the context of the workforce capacity challenges experienced during the COVID-19 pandemic. This study seeks to describe practical rather than strictly theoretical insights per se, and it is therefore intended for health policymakers, public health practitioners, and researchers considering innovative and flexible approaches for addressing short-term workforce capacity issues during the COVID-19 pandemic as well as other health emergency situations.

## 2. Methods

### 2.1. Management and Funding

**Figure 3:**
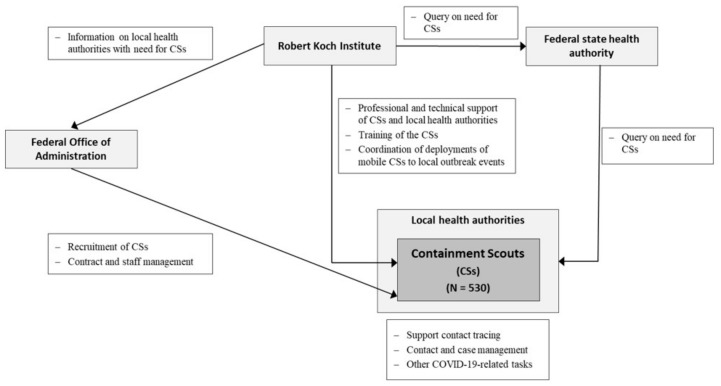
Containment Scout Initiative: Recruitment and management.

The RKI and the BVA are responsible for the management of the CSI (Figure 3). The RKI provides professional and technical support to the Containment Scouts (CSs) and local health authorities, and it organises and coordinates the CSs’ deployment with the federal state health authorities and local health authorities (Figure 3). As the employer, the BVA is in charge of staffing aspects, such as recruitment and contract management (Figure 3).

The German Federal Ministry of Health (Bundesministerium für Gesundheit, BMG) funds the CSI. The project proposal was submitted in March 2020 and was initially approved until the end of 2020. The total cost for the first phase amounted to approximately 11.3 million Euros (~13.5 million US$), which funds the CSs (530 positions) as well as six employees at the RKI.

### 2.2. Project Participation and Coordination

Of the federal states in Germany, 15 out of 16 participated in the first phase of the CSI. Prior to starting recruitment, local coordinators at each federal state health authority were contacted to assess the need and the distribution for CSs in their local health authorities. The theoretical number of CSs available per federal state was determined by population size. Recruitment started in Bavaria, Baden-Wurttemberg, and North Rhine-Westphalia due to their high COVID-19 infection rates.

### 2.3. Containment Scouts: Job Description, Job Profile, and Training Materials

The job advertisements were published widely on 20 March 2020 via university mailing lists, as well as through social media and news websites. The CS positions were paid 2325 Euro per month before tax (2736 US$) and were advertised as a six-month, full-time job. The BVA conducted the selection process and telephone interviews. The suitability of the applicants was assessed based on professional backgrounds, which were grouped into four categories. The intention was to predominantly recruit people with a medical or health-related background (termed category 1 and 2, respectively). The applicants’ suitability was also assessed by their level of motivation, communication skills, stress tolerance, and ability to work in a team. The local health authorities have been allowed to fill CS positions with their own applicants since June 2020 so that they can effectively fill vacancies in case any of the CSs terminate their employment early. The CSs received online training material before starting their work in the local health authorities. The training materials were developed and provided by the RKI, alongside materials from the Federal Association of Physicians of German Public Health Departments and companies that provide reporting software to the local health authorities. The materials included the following main topics: (1) Introduction to infectious disease epidemiology, (2) COVID-19-related outbreak investigations and contact tracing, (3) Reporting software, and (4) Introduction to the public health system in Germany. The CSs received continuous professional support from the RKI during their time of employment through different forms of contact, including email, telephone, and video conference calls.

### 2.4. Mobile Containment Scouts

In addition to the 500 regular CSs, 30 mobile Containment Scouts (mCSs) positions were created in June 2020 and recruited from the BVA applicant pool to provide a quick response to support health authorities with local outbreaks. Local health authorities have been able to contact the RKI for further support, which responds by sending out a team of two to six mCSs within a couple of days and for a maximum of three weeks at a time. The mCSs keep track of infection chains in regions with temporarily high COVID-19 infections rates, particularly through contact tracing.

### 2.5. Surveys: First Assessments of the CSI

The RKI conducted two surveys among the CSs and one among the participating local health authorities in order to assess the first phase of the CSI. In the first survey (May 2020), all the CSs employed at that time (N = 494) were asked to provide information on their professional background and their student status. In June 2020, all participating local health authorities (N = 265) were asked to inform about the CSs’ tasks and assess the CSs’ professional backgrounds and performance in the second survey. Furthermore, they were asked to provide their interest in an extension of the CSI as well as any general comments. The third survey (September 2020) was sent to all CSs with current or former contracts (N = 578). It aimed to understand the CSs’ willingness to continue working for the public health sector as well as any reasons for leaving the CSI. The three surveys were sent to the intended respondents via E-Mail and made available with the survey administration software Google Forms. In line with data protection, all responses were stored in a secure location with access limited to the RKI coordination team only.

## 3. Results

### 3.1. Recruitment of the CSs and Continuous Exchange between Project Participants

The first CSs started their employment in April 2020, and more than 500 CSs had been recruited in total after ten weeks. The actual number of employed CSs fluctuated due to regular recruitment and terminations of employment. Despite these fluctuations, more than 500 CSs worked fairly consistently at any one time in almost 270 of the 380 local health authorities in Germany during the first phase of the CSI (March 2020 to October 2020).

The close cooperation of different institutions at the national, state, and local level enabled a clear division of responsibilities, including project management and professional support (RKI), recruitment and contract management (BVA), funding (BMG), allocation of CS positions based on local need (federal state health authorities) and the implementation of COVID-19-related tasks (local health authorities).

### 3.2. Professional Background and Further Perspectives

Of the 494 CSs recruited at the time of conducting the first survey, the majority (57.9%, 286/494) provided information on their professional background (Table 1). Among these, approximately one third (36.0%, 103/286) had graduated or were currently a student in a medical or health subject (category 1), 8.4% (24/286) had a professional health-related background, such as trained nurses and paramedics (category 2), and 53.1% (152/286) had graduated or were currently studying in non-health-related subjects (category 3) (Table 1). 

Among all CSs, the proportion of current university students was 50.7% (145/286), and, of those, 5.5% (8/145) were medical students. In the third survey, about one third of those who provided reasons for leaving the CSI (35.7%, 45/126) stated that they left or planned to leave the project since they were directly hired by the local health authorities. Another near third (32.5% 41/126) returned to their studies or entered university. Almost a quarter (24.6%, 31/126) accepted other job offers, and a small number (17.5%, 22/126) did not seek to extend their contracts due to other reasons. In total, 66.0% (128/194) of the CSs reported a willingness to continue working in the public health sector.

### 3.3. Continued Professional Support

The training materials provided to the CSs were updated at the end of the first phase to reflect new recommendations for contact tracing and contact management in Germany. To ensure the contact tracing procedures were up to date, the CSs were directed to the RKI website, where recommendations were updated almost weekly, and the RKI hosted three online meetings on the topic. The questions posed by the CSs in the first phase of the CSI ranged from how to determine contacts and how to carry out contact case management, to the role of the German contact tracing app (“Corona Warn App”) (Table 2).

### 3.4. Task and Performance Assessment

The response rate for the second survey, which was conducted among local health authorities, was 80.4% (213/265). According to respondents, 96.7% (206/213) of the CSs exclusively or predominantly worked on COVID-19-related tasks. Of these, the tasks most often performed were contact tracing (86.9%, 185/213), electronic documentation of COVID-19 cases (81.2%, 173/213), and support of telephone services relating to COVID-19 (64.3%, 137/213). A total of 39.4% (84/213) of respondents reported that the CSs helped with the organisation of testing for SARS-CoV-2.

The local health authorities rated the professional qualifications of the CSs for the assigned tasks variably: 17.3% (33/191) rated the professional qualifications as “very good”, the majority (32.5%; 62/191) as “good”, 30.9% (59/191) as “sufficient”, and 19.4% (37/191) as “unsatisfactory”. Frequently reported critique in the comment section was the lack of professional expertise/background and experience in medicine or health. Regarding the CSs’ general performance, the majority (60.5%; 121/200) of respondents rated it as “very good” and 35.0% (70/200) as “good”. Only 4.5% (9/200) of the local health authorities assessed it as “sufficient”, and none were rated as “unsatisfactory”. Because data on performance and background were not available at the individual level, we were unable to assess the difference in performance between CSs with a medical or health-related background and those without.

### 3.5. Support of the Mobile Containment Scouts

The mCSs were based at the local health authorities in Berlin (mCSs = 18) and at the RKI (mCSs = 5). Between June 2020 and October 2020, the mCSs supported nine local health authorities in four federal states for 125 days in total. The average length of support was 14 days, and the average team size was 4 mCSs. All of the local health authorities needed additional support to contain outbreaks, conduct mass testing, or replace staff who had been lost at short notice due to relocation or sickness.

### 3.6. Project Extension

Almost all local health authorities (99.5%, 201/202) were interested in extending the CSI beyond the original project duration. The BMG approved the first extension of the CSI in August 2020 to support the local health authorities a few months later throughout the winter season in 2020/2021, when higher infection rates were expected. In February 2021, the BMG approved the second project extension until March 2022 during the third wave in Germany.

## 4. Discussion

This article describes the first phase of the newly developed CSI and the substantial efforts to hire, train, deploy, and support qualified staff to local health authorities in urgent need of additional staff during the COVID-19 pandemic.

There has been a substantial need for additional contact tracing staff at short notice worldwide, including in most European countries [13,14]. As a result, various measures to increase the health workforce for contact tracing have been implemented [7,15,16]. These include the use of call centres (France and UK), recruitment of retired medical doctors and nurses (UK), and volunteers (Cyprus) [17]. Medical or other health-related students have also been used for contact tracing staff in many countries [15,17]. In the USA, several academic public health programmes and schools trained students as contact tracers in response to COVID-19 [18]. Community-based partnerships between universities and local health departments were established to compensate for workforce shortages [19,20,21]. These programmes were mostly volunteer-based [19,20,22,23], and students received academic credit and work experience in return [20,22]. In one example, a funding opportunity enabled a programme to pay the contact tracers [19]. All programmes found in the literature provided training and were targeted at students with a background in public health or other health subjects [19,20,22]. The workforce has also been upscaled in previous emergency situations, such as for Ebola in West Africa, including using volunteers to carry out contact tracing [24].

We were unable to assess the difference in performance after training between CSs with a health-related background and those without. However, the CSI indirectly indicates that medical and health-related backgrounds may not be necessary for this initiative because the majority of recruited CSs (55.5%) did not fulfil these criteria and the overall work performance of the CSs was rated as very good or good by over 90%. This may indicate that social and other skills are contributing factors for successfully carrying out contact tracing, supported also in the literature [23,25]. Some training should still be provided to prepare the CSs. Therefore, they received training materials on COVID-19 and the basics of epidemiology at the start of their job, as recommended [25] and carried out by other contact tracing programmes [22,26]. Our findings suggest that the training provided to the CSI is sufficient, which addresses the questions of Rajan et al. [17] on whether Germany has a sufficiently high proportion of experienced contact tracers.

The CSI has some strengths compared to other programmes. Firstly, by providing a salary and contracts of at least six months to the CSs, the initiative offers longer-term support compared to programmes based on volunteers. Volunteers can be challenging to maintain over time [26], particularly when paid programmes run parallel with voluntary activities [20]. Therefore, it is expected that a large proportion of volunteers return to their original activities after a while, as reported in the USA [26]. Secondly, because the CSI is not just limited to students, the pool of suitable applicants was much larger. The CSs are supposed to work full-time, thereby offering substantial support to local health authorities. Thirdly, the CSs’ support also extends beyond the duration of the project. Many local health authorities were able to offer qualified CSs longer-term contracts, as well as better positions with higher salaries. Fourthly, the CSI was coordinated by statutory bodies and integrated into the statutory public health system, allowing it to be implemented nationwide, in contrast to programmes that are limited to local or regional areas and not integrated into a national strategy.

The CSI, some of its features, and what are identified above as strengths of the CSI may not be directly transferable or applicable to other settings, such as countries that do not have strong statutory public health systems, where funding cannot be sustained and centralised initiatives are not possible. The CSI could, however, be adapted to other settings, as was done from the Chinese approach for Germany, taking into account the structures of the system, funding, culture, and other relevant health system factors.

The support of almost all local health authorities of the CSs and their strong interest in an extension of the CSI indicates that it has been an effective short-term public health measure to sustain and strengthen the local health authority workforce during the COVID-19 pandemic. It is also one example of how the COVID-19 pandemic has presented a unique opportunity for learning from similar approaches and transferability, innovation through adaptation to a novel setting, and rapid action and scale up in public health. For example, the health system in Germany is federally organised. A central payment by the Federal Ministry of Health to support local health authorities is therefore unusual and indicates the adaptation of the system to extraordinary circumstances.

However, the CSI is not designed to meet all the needs for additional staff in the long term. The workforce challenges in the German public health system in general and particularly in the local health authorities are structural in nature and go far beyond the need for contact tracing staff. In order to address some of these issues, the German Government announced the “Pact for the Public Health Service” (Pakt für den Öffentlichen Gesundheitsdienst) in September 2020. This includes providing an extra four billion Euros and developing a new plan to increase staffing and improve the digital infrastructure for public health services until the end of 2022 [27]. Almost a year after the announcement of the pact, the implementation of the agreements is still delayed [28]. Although the local health authorities have hired a sizeable number of CSs, the CSI alone cannot compensate for the structural deficits relating to the workforce challenges that still exist in the local health authorities. The pandemic and the CSI have highlighted the importance of comprehensively and sustainably strengthening the German public health system.

This article provides a first description as well as some initial insights into the CSI in order to help with planning for contact tracing in the future and in other settings. It has several limitations, which could be addressed in further studies. For example, the CSs’ experience and the context in which they worked could not be compared between the 16 German states; it was not possible to explicitly compare performance between CS’ with/without health-related backgrounds; and it was not possible to assess the impact and effectiveness of contact tracing as a whole. This study also offers practical rather than strictly theoretical insights, which could be a valuable area for future study.

## 5. Conclusions

The CSI is an example of how short-term solutions to address critical health system capacity issues can be quickly integrated into the public health system and implemented nationwide, as well as how initiatives can be adapted to different settings. This particular approach has been successful in temporarily increasing workforce capacities in Germany and has been extended to meet continued need. However, it is an interim solution and highlights the need for longer-term and sustainable funding and strengthening of the public health system in Germany.

## Figures and Tables

**Table 1:** Profession backgrounds of the Containment Scouts (first phase).

Category	Level of Experience or Knowledge	Subject Area	%	N
1	Graduated or current student in medical or health subjects	e.g., human medicine, public health, health management	36.0	103
2	People with health-related professional background	e.g., trained (health) nurses, paramedics	8.4	24
3	Graduated or current student in non-health-related subjects	e.g., sociology, pedagogy, sports science	53.1	152
4	Not graduated or current student, or no professional health-related background	e.g., office administrator	2.4	7

N = 286.

**Table 2:** Selection of questions from Containment Scouts to the Robert Koch Institute (first phase).

Categorisation of contacts of SARS-CoV-2 positive cases	Could the CT value of SARS-CoV-2 cases be considered for the assessment of contact person categorisation?
	Dealing with choir rehearsals indoors or outdoors—In case of a COVID-19 infection, should everyone who participated in the choir rehearsal be considered a contact person?
	Do both criteria (distance <1.5m and 15 min and longer contact) have to be met to fulfil quarantine requirements or is one criterion already sufficient?
	People who have been classified as category I contact person and have tested negative for SARS-CoV-2 must remain in quarantine for 14 days after the last contact with the confirmed case. Does the last contact with the confirmed case count as day 1 of quarantine?
Case management	Should previous cases be reclassified as contacts after repeated exposure?
	How does one deal with cases that remain positive over a long period of time?
Contact categorisation and case management of medical staff	The hospital has several employees who, after having a SARS-CoV-2 infection, now have SARS-CoV-2 antibodies (Roche test). To what extent can this be included in the contact person categorisation?
	How are medical staff categorised without adequate protective equipment but with simple face masks?
Infectiousness	When do people who are positive for SARS-CoV-2 become infectious?
	For a few weeks, I have observed that in families with members who are positive for SARS-CoV-2, all family members fall ill on most occasions. This was not the case in spring/summer. Is it possible that the virus has changed and has therefore become even more contagious?
Travellers returning from risk areas	What is the procedure for people who return from countries where infection rates are not known/not credible?
	How does one categorise people who return from travel abroad who had contact with people who tested positive for SARS-CoV-2 in a double-decker bus?
Corona Warn App	Should people who receive a high-risk-alert by the Corona Warn App (i.e., contact with an infected person) be tested for SARS-CoV-2? If so, will the RKI adapt its recommendations accordingly?

## Data Availability

Not applicable.
